# Bio-imaging: late gadolinium enhancement in dilated cardiomyopathy and its relation to novel biomarkers of fibrosis and remodeling

**DOI:** 10.1186/1532-429X-15-S1-P146

**Published:** 2013-01-30

**Authors:** Hassan Abdel-Aty, Evangelos Giannitsis

**Affiliations:** 1 Department of Cardiology, University of Heidelberg, Heidelberg, Germany

## Background

Focal myocardial fibrosis is frequently observed in dilated cardiomyopathy (DCM) and predicts future major adverse cardiovascular events and patient outcome. However, the pathogenesis of fibrosis in DCM remains poorly understood. Novel serum biomarkers of fibrosis such as Endoglin have been recently identified as a trigger of myocardial fibrosis in heart failure. Accordingly, we sought to investigate the relationship between myocardial tissue fibrosis as identified by non-invasive late gadolinium contrast enhancement (LGE) CMR and serum biomarkers of fibrosis/ remodeling in a cohort of DCM patients.

## Methods

We investigated 80 DCM patients (50 ± 16 years, 60 males) with known DCM using a 1.5T MRI scanner (Philips Achieva). Short axis slices covering entirely both ventricles were acquired using a regular SSFP-sequence to measure ventricular volumes and ejection fraction. Focal myocardial fibrosis was assessed with LGE CMR images acquired 10 minutes after i.v. injection of 0.2 mmol/kg Gd-DTPA (Magnevist). SSFP and LGE CMR images were assessed by different observers who were blinded to CMR measurement results and clinical data. Using dedicated scar software (Philips Viewforum) LGE CMR lesions were drawn manually on all short axis images and quantified as percent of the total LV mass. The extent of LGE CMR was correlated to serum markers of fibrosis (Endoglin), myocardial necrosis (high sensitivity Troponin T (hs-TnT)) and heart failure (NT pro-BNP).

## Results

LGE CMR lesions were present in 33 patients (41%) with an average extent of 4±4% and were predominantly visualized (48%) intra-murally within the septum.

Serum Endoglin and hs-TNT levels were significantly elevated in LGE CMR patients (6.7±3 ng/ml vs. 5.3±2 ng/ml and 28.3±46 pg/ml vs. 10.7±15 pg/ml; p < 0.02 and 0.04. Only serum levels of Endoglin (area under the curve (AUC): 0.64; p<0.04) and hs-TnT (AUC:0.66; p<0.02) but not NT pro-BNP predicted the presence of LGE CMR.

## Conclusions

We provide first evidence that novel serum markers of interstitial cardiac fibrosis and myocardial necrosis are elevated in DCM patients with myocardial fibrosis detected on LGE CMR. The relation between Endoglin and LGE CMR lesions is especially important, since it provides novel insights into the pathogenesis of myocardial fibrosis in DCM patients linking interstitial cardiac fibrosis and markers of smoldering continuous myocardial necrosis to the dys-regulated renin-angiotensin-system which, in turn, is activated and modulated by serum Endoglin.

## Funding

none

